# Acute acetaminophen ingestion improves performance and muscle activation during maximal intermittent knee extensor exercise

**DOI:** 10.1007/s00421-017-3794-7

**Published:** 2018-01-13

**Authors:** Paul T. Morgan, Joanna L. Bowtell, Anni Vanhatalo, Andrew M. Jones, Stephen J. Bailey

**Affiliations:** 1 Sport & Health Sciences, University of Exeter, Exeter, UK; 2 Present Address: School of Sport, Exercise and Health Sciences, Loughborough University, Leicestershire, UK

**Keywords:** Analgesic, Critical torque, Electromyography, Neuromuscular fatigue, Single-leg exercise

## Abstract

**Aim:**

Acetaminophen is a commonly used medicine for pain relief and emerging evidence suggests that it may improve endurance exercise performance. This study investigated some of the physiological mechanisms by which acute acetaminophen ingestion might blunt muscle fatigue development.

**Methods:**

Thirteen active males completed 60 × 3 s maximum voluntary contractions (MVC) of the knee extensors with each contraction separated by a 2 s passive recovery period. This protocol was completed 60 min after ingesting 1 g of maltodextrin (placebo) or 1 g of acetaminophen on two separate visits. Peripheral nerve stimulation was administered every 6th contraction for assessment of neuromuscular fatigue development, with the critical torque (CT), which reflects the maximal sustainable rate of oxidative metabolism, taken as the mean torque over the last 12 contractions. Surface electromyography was recorded continuously as a measure of muscle activation.

**Results:**

Mean torque (61 ± 11 vs. 58 ± 14% pre-exercise MVC) and CT (44 ± 13 vs. 40 ± 15% pre-exercise MVC) were greater in the acetaminophen trial compared to placebo (both *P* < 0.05). Voluntary activation and potentiated twitch declined at a similar rate in both conditions (*P* > 0.05). However, the decline in electromyography amplitude was attenuated in the acetaminophen trial, with electromyography amplitude being greater compared to placebo from 210 s onwards (*P* < 0.05).

**Conclusion:**

These findings indicate that acute acetaminophen ingestion might be ergogenic by increasing CT and preserving muscle activation during high-intensity exercise.

**Electronic supplementary material:**

The online version of this article (10.1007/s00421-017-3794-7) contains supplementary material, which is available to authorized users.

## Introduction

Neuromuscular fatigue is defined as a decrease in skeletal muscle force production capacity (Gandevia 2001). This neuromuscular fatigue development can arise from physiological perturbations within the central nervous system, termed central fatigue, or within, or distal to, the neuromuscular junction, termed peripheral fatigue (Gandevia 2001). Exercise-induced fatigue is a complex, multi-factorial process, the physiological bases of which are still widely debated (Enoka and Duchateau 2008; Gandevia 2001; Hureau et al. 2016; Place et al. 2010; Taylor et al. 2016). Indeed, recent research suggests that peripheral fatigue and central fatigue develop inter-dependently and are likely to interact in a coordinated manner to determine neuromuscular fatigue development (Hureau et al. 2016).

Following the onset of muscle contractions, group III and IV afferents discharge in response to mechanical and metabolic stimuli, contributing to the sensation of muscle pain during exercise (O’Connor and Cook 1999; McCord and Kaufmann 2010; Pollak et al. 2014). Group III/IV muscle afferent feedback, and the associated sensation of pain, appears to play a role in neuromuscular fatigue development through modulating both central and peripheral fatigue. Indeed, when the ascending projection of group III and IV muscle afferents is attenuated via intrathecal fentanyl administration, central motor drive is increased (as inferred via electromyography, EMG) and peripheral fatigue development is expedited (Amann et al. 2009, 2011; Blain et al. 2016). Therefore, interventions that can modulate skeletal muscle pain sensation have the potential to impact muscle activation and exercise-induced fatigue development.

Acetaminophen (ACT; or paracetamol) is a commonly used non-prescription pain reliever which is considered one of the safest non-opioid analgesics at therapeutic doses (Toussaint et al. 2010). Acute ACT consumption has been shown to improve exercise performance concomitant with a lower pain sensation (Foster et al. 2014; Mauger et al. 2010). The mechanisms that underlie the analgesic effect of ACT are not completely understood, but are considered to be predominantly mediated by central factors (Anderson 2008; Graham et al. 2013; Smith 2009; Toussaint et al. 2010). Conventionally, the analgesic effect of ACT has been attributed to the inhibition of cyclooxygenase enzymes, which stimulate nociceptor discharge through the synthesis of prostaglandins (Graham et al. 2013; Jóźwiak-Bębenista and; Nowak 2014). There is also evidence that the analgesic effect of ACT is linked to potentiation of descending serotoninergic pathways (Pickering et al. 2006, 2008), and to modulation of opioid and cannabinoid receptors (Graham et al. 2013). These mechanisms likely interact to lower pain sensation after acute ACT ingestion by increasing the nocioceptive stimuli required to evoke a given pain sensation. Although ACT ingestion has been reported to increase resting cortico-spinal excitability, as inferred from an increased motor evoked potential amplitude (Mauger and Hopker 2013), the neuromuscular bases for blunted exercise-induced fatigue development following ACT ingestion have yet to be investigated.

In addition to altering pain sensation and neuromuscular function, ACT ingestion might attenuate fatigue development and enhance performance by modulating aspects of the power or torque-duration relationship. The asymptote of the hyperbolic torque-duration relationship is termed the critical torque (CT) and can be estimated from the mean torque produced over the last 12 maximum voluntary contractions (MVCs) of a 60 MVC knee extension protocol (Burnley 2009). The CT reflects the maximal sustainable rate of oxidative metabolism (Jones et al. 2008, 2011 for review) and represents a critical threshold for neuromuscular fatigue development (Burnley et al. 2012). The curvature constant of the torque-duration hyperbola is termed the *W*′ and represents a fixed amount of torque-impulse (surrogate measure of ‘total work’) that can be completed above CT (Burnley and Jones 2016; Jones et al. 2008; Poole et al. 1988). Together, CT (critical power or critical speed for other forms of exercise) and *W*′ (or its equivalent) can be used to accurately predict exercise performance (Black et al. 2014; Burnley et al. 2012). An intervention that enhances high-intensity exercise tolerance or performance, or attenuates fatigue development, would be expected to enhance CT or *W*^′^ (or their exercise modality-specific equivalents). For example, critical power, but not *W*^′^, is increased following endurance training concomitant with improved endurance exercise performance (Vanhatalo et al. 2008). Therefore, the reported improvement in exercise performance with acute ACT ingestion (Foster et al. 2014; Mauger et al. 2010, 2014) would be expected to be linked to improvements in CT and/or *W*′, but this has yet to be investigated.

The purpose of this study was to test the hypotheses that, compared to placebo, acute consumption of 1 g ACT would reduce the rate of neuromuscular fatigue development, and increase total impulse (torque-time integral), CT and muscle activation during a 5 min single-leg intermittent MVC test.

## Materials and methods

### Participants

Thirteen healthy male volunteers (mean ± SD: age 31 ± 7 years, height 1.76 ± 0.08 m, body mass 75 ± 11 kg) provided written, informed consent to participate in the present study, which was approved by the Ethics Committee of Sport and Health Sciences (University of Exeter). After being informed of the experimental procedures and associated risks, all participants completed a medical health questionnaire to ensure they could safely consume ACT prior to performing exhaustive exercise. Subjects were not consumers of any ‘pain relief’ medication (prescription or non-prescription) over the course of the study. None of the subjects had a history of motor or neurological disorders.

### Experimental design

Subjects visited the laboratory on three occasions over a 3- to 4-week period with all tests separated by at least 1 week and conducted at a similar time of day (± 90 min) to limit changes in quadriceps strength and to account for diurnal variations in neuromuscular excitability. The first laboratory visit was used to familiarise subjects to the measurements and experimental protocol described below. During these sessions, the settings and placement of the dynamometer and EMG and peripheral nerve stimulation electrodes were recorded for each subject. Subsequently, subjects performed the fatiguing protocol under two conditions (see ‘Experimental protocol’): placebo (PL) and ACT.

### Experimental protocol

Subjects completed a preliminary trial for familiarisation to the measurement techniques and experimental protocol. An isokinetic dynamometer (Biodex System 3, Shirley, NY, USA) was used in all tests and adjusted so that the axis of rotation of the lever arm was in line with the lateral epicondyle of the right femur. Subjects were seated with the hip and knee joints at relative angles of 155° and 90°, respectively. The remainder of the chair settings were recorded and replicated in all subsequent trials to ensure an identical body position was assumed throughout the experimental trials. The semi-supine position was employed as it permitted better access to the superficial femoral nerve for peripheral nerve stimulation as verified during pilot testing. Inelastic padded Velcro straps were fastened at the ankle, quadriceps, hip and shoulders to maintain a stable body position. The procedures adopted during the familiarisation trial were replicated in all experimental trials.

**Fig. 1:**
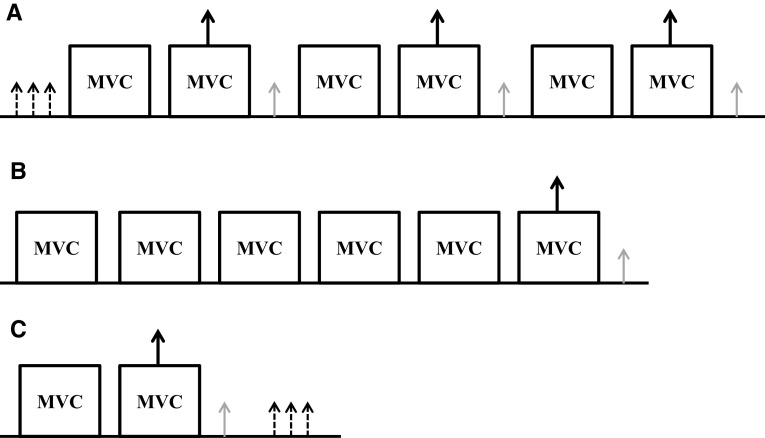
Schematic of the procedures used prior to (panel **a**), during (panel **b**) and within 10 s following (panel **c**) the 60 maximal isometric voluntary contraction (MVC) protocol. 10 s separated each single pulse stimulation administered at rest (small dashed arrows). **a** (pre) **c** (post) 45 s rest period separated maximal efforts (MVCs). Single pulse stimuli were administered during peak force production of MVCs (large solid arrow) and immediately (1–2 s post; small grey arrows). **b** 60 MVC protocol of the knee extensors. The figure presents a period of 30 s which is repeated sequentially for 5 min. Each MVC was held for 3 s and interspersed by a 2 s passive recovery period. Every 6th MVC was accompanied by single pulse stimuli administered during peak force production (large solid arrow) and immediately following (< 2 s post; small grey arrows). This cycle was repeated 10 times such that the protocol spanned 5 min requiring the completion of 60 MVCs. MVC, maximal voluntary isometric contraction. Surface electromyography (EMG) was measured throughout

The trials on visits 2 and 3 were completed in a double-blind, randomised fashion using a cross-over experimental design. Prior to each visit, subjects were required to refrain from caffeine (for at least 12 h), strenuous exercise and alcohol (for at least 24 h), analgesics and any form of anti-inflammatory drug (for the duration of the experimental trial) and to arrive in a fully rested and hydrated state. Subjects were instructed to maintain their usual diet and exercise regime during the study. 1 g of maltodextrin (placebo) or 1 g of acetaminophen was ingested orally, 60 min prior to the exercise bout such that the start of the exercise trial was expected to coincide with attainment of the peak plasma [acetaminophen] (Anderson et al. 2008; Forrest et al. 1982). Following oral ingestion, ACT is rapidly absorbed from the gastrointestinal tract and its bioavailability ranges from 70 to 90% (Forrest et al. 1982). The trials started with a standardised isometric warm-up routine (10 isometric contractions for 3 s at 50% of pre-exercise MVC as determined during familiarisation testing) and testing of the optimal EMG electrode, anode and cathode placement and stimulation intensity for peripheral nerve stimulation. Neuromuscular function was assessed pre-trial, during and (< 10 s) post-trial. Single peripheral nerve stimulation pulses were manually triggered at rest to determine pre-exercise neuromuscular function, namely the characteristics of the M-wave response (M-wave amplitude; *M*_max_) to supramaximal nerve stimulation, contractility of the muscle (i.e., maximal rate of force development, half-relaxation time and contraction time), voluntary activation (VA) and potentiated twitch force (pTw). During MVCs, peripheral nerve stimulation pulses were triggered to occur as soon as a peak torque was achieved (typically 1.5 s into a 3 s contraction), each separated by a 40 s rest. The stimuli were also delivered 1–2 s after the cessation of the contraction to provide a resting twitch (rTw). Identical measurements were repeated as soon as possible (< 10 s) after the fatiguing exercise to determine post-exercise neuromuscular function (see Fig. 1).

The intermittent isometric contraction protocol used in this study had a similar design to that presented by Burnley (2009). The protocol consisted of repeated brief MVCs (3 s contraction, 2 s rest), timed via a visual prompt to ‘go’ and ‘relax’ (separate to the torque output screen), accompanied by the same verbal instructions from the experimenter. The protocol was terminated when the subjects completed the 60th MVC. Every 6th contraction was accompanied by peripheral nerve stimulation during and post-MVC (as described for pre- and post-trial measurements). Subjects were not given a visual representation of the torque produced during each MVC or made aware of the number of MVCs elapsed during the protocol. Subjects were instructed to continue to perform maximal contractions throughout. The coefficient of variation for MVC performance (mean torque through 60 MVCs) and electromyography root mean square (EMG_RMS_) amplitude using this experimental set up was 2.9% and 4.8%, respectively as calculated during pilot testing.

### Measurements

#### Torque

Knee extensor torque from the Biodex isokinetic dynamometer was sampled at 1000 Hz and low-pass filtered at 40 Hz, before being displayed on a wide screen monitor using Spike2 (CED, Cambridge, UK). Torque was expressed throughout as a percentage (%) of pre-exercise MVC.

### Electromyography

Surface EMG activity was recorded from vastus lateralis, vastus medialis and rectus femoris of the quadriceps and biceps femoris of the hamstring of the right leg using active bipolar bar electrodes in a single differential configuration (DE2.1, DelSys Inc, Boston, MA, USA). The electrodes were placed over the respective muscle bellies (SENIAM guidelines). Double-sided adhesive tape and a hypoallergenic medical tape were used to ensure the EMG sensor stability for recording electrodes. The skin area underneath each EMG electrode was shaved, then exfoliated and cleaned with alcohol to minimise the skin impedance. The EMG and torque signals were pre-amplified (1000×), band-pass filtered (20–450 Hz, Bagnoli-8, DelSys Inc, Boston, MA, USA), and then transferred to a computer with a sampling frequency of 2 kHz and high-pass filtered at 10 Hz. EMG and torque data were recorded continuously and digitised synchronously with 16 bit resolution via an A/D converter (± 5 V range, CED 1401 power, Cambridge, UK). The electrodes were used to record: (1) evoked muscle action potential (peak-to-peak amplitude of the M-wave); and (2) EMG to estimate muscle activity and the output of spinal motoneurons (motor unit recruitment and firing frequency). EMG was average rectified using the root mean square method (EMG_RMS_). EMG_RMS_ was then normalised to the pre-exercise maximum (or maximal EMG signal) and the local M-wave amplitude (closest measure of the M-wave to the MVC) to exclude any changes to the EMG trace to changes in local excitability. The ground electrode was placed over the patella of the right leg.

### Peripheral nerve stimulation

Electrical stimulation was applied by a constant current stimulator (Digitimer Stimulator DS7, Digitimer, UK). M-waves were elicited by supramaximal percutaneous electrical stimulation of the femoral nerve (200 µs duration). The cathode was placed over the femoral nerve in the inguinal fossa, approximately 3–5 cm below the inguinal ligament in the femoral triangle. Once the M-wave was elicited, the maximum amplitude (peak-to-peak) of the M-wave was determined (*M*_max_) for the vastus lateralis and vastus medialis. To determine the stimulation intensity (current), single stimuli were delivered in 20 mA step-wise increments from 100 mA until a plateau in quadriceps rTw and M-wave were observed. To ensure a supramaximal response, the current was increased by an additional 30% (mean ± SD current = 194 ± 81 mA; Burke 2002; Goodall et al. 2010; Neyroud et al. 2014). The average *M*_max_ was obtained from three stimuli, with ~ 8–10 s separating each pulse at rest. Peak torque, maximal rate of force development, half-relaxation time and contraction time were analysed for all rTw and pTw.

### Data analyses

Data were analysed using a custom written script developed in Spike2 software (CED, Cambridge, UK). Mean torque for each 3 s contraction was determined for all tests as the mean value over a 1 s period which approximated the plateau level of the highest torque (i.e., 500 ms before and after the peak torque). The pTw was calculated as the peak torque achieved following the single pulse delivered 1 s post-MVC. The twitch torque superimposed onto the peak force production of the MVC (sTw) was calculated as the increment in torque immediately following the pulse during MVCs. The torque-impulse was calculated as the area under the torque-time curve by accumulating the time integral of each MVC (3 s) by the difference in torque between MVCs. The end-test torque (i.e., CT) during the 60 MVC test was defined as the mean of the last 12 contractions (i.e., the last 60 s; Burnley 2009). The *W*′ was calculated as the area above the CT from the torque-time curve (i.e., impulse above CT). Central fatigue was assessed as the maximal voluntary activation of the motoneuron pool (VA, %), calculated using the interpolated twitch method from peripheral nerve stimulation (Merton 1954). Specifically, the increment in torque evoked during the MVCs was expressed as a fraction of the amplitude of the potentiated twitch produced with the same stimulus in the relaxed muscle post-MVC. The level of voluntary drive was then quantified as a percentage: [1 − evoked torque (superimposed on voluntary torque, sTw)/ (potentiated twitch torque, pTw) × 100] (i.e., Allen at al. 1998).

The changes in maximal voluntary torque, rTw, pTw, VA and EMG_RMS_, were used to quantify peripheral fatigue and central fatigue. The maximal EMG was taken from the first MVC during the 60 MVC task and compared to the last MVC at task end. The neuromuscular parameters extracted from the three sets of maximal contractions completed post-exercise were tested for statistical differences between sets of contractions and then compared to the first set of MVCs completed pre-exercise (Froyd et al. 2013; Pageaux et al. 2015; Doyle-Baker et al. 2017). Neuromuscular function was also measured for each of the stimulated contractions during the exercise and normalised to the corresponding pre-exercise values at 100% MVC. All neuromuscular parameters and torque were averaged across the protocol using 6 (30 s) bin averages.

### Statistics

Paired-samples *t*-tests were used to compare the mean torque, total impulse, CT and *W*^ʹ^ (i.e., impulse above CT) between ACT and PL conditions. In addition, paired-samples *t*-tests were used to assess parameters of neuromuscular function at task end between trials including rTw, pTw, maximal rate of force development, half-relaxation time and contraction time. The profiles of torque, VA, pTw and M-wave amplitude were analysed using two-way ANOVAs with repeated measures (using 12 contraction averages; i.e., 6 time points). Normalised EMG_RMS_ were analysed using two-way ANOVAs with repeated measures using 30 s mean values (i.e., 10 time points). Where the ANOVA revealed a significant interaction effect, post hoc tests were completed using a Bonferroni correction. For calculation of effect size, partial eta squared (*η*^2^) was used for omnibus tests. Cohen’s d was used to calculate the effect size for paired t-tests and post hoc comparisons. A *t*-test was also conducted on the differences in EMG and force production (torque) from task end (i.e., 300 s) to 150 s (mid-point) to assess the rate of change as the protocol progressed. All statistical tests were performed both on % change and raw data. Where sphericity was violated, a greenhouse-geisser correction factor was used. For all the tests, results were considered statistically significant when *P* < 0.05. Data are presented as mean ± SD unless otherwise indicated. All statistical analyses were conducted using IBM SPSS Statistics version 23.

## Results

The mean MVC torque achieved prior to the 60 MVC protocol was 232 ± 47 and 228 ± 48 N m for PL and ACT, respectively. VA of the knee extensors achieved during the preliminary MVCs was 88 ± 7% and 87 ± 5 for PL and ACT, respectively. Baseline MVC and VA were not different between conditions (*P* > 0.05).

### 60 MVC performance

**Fig. 2:**
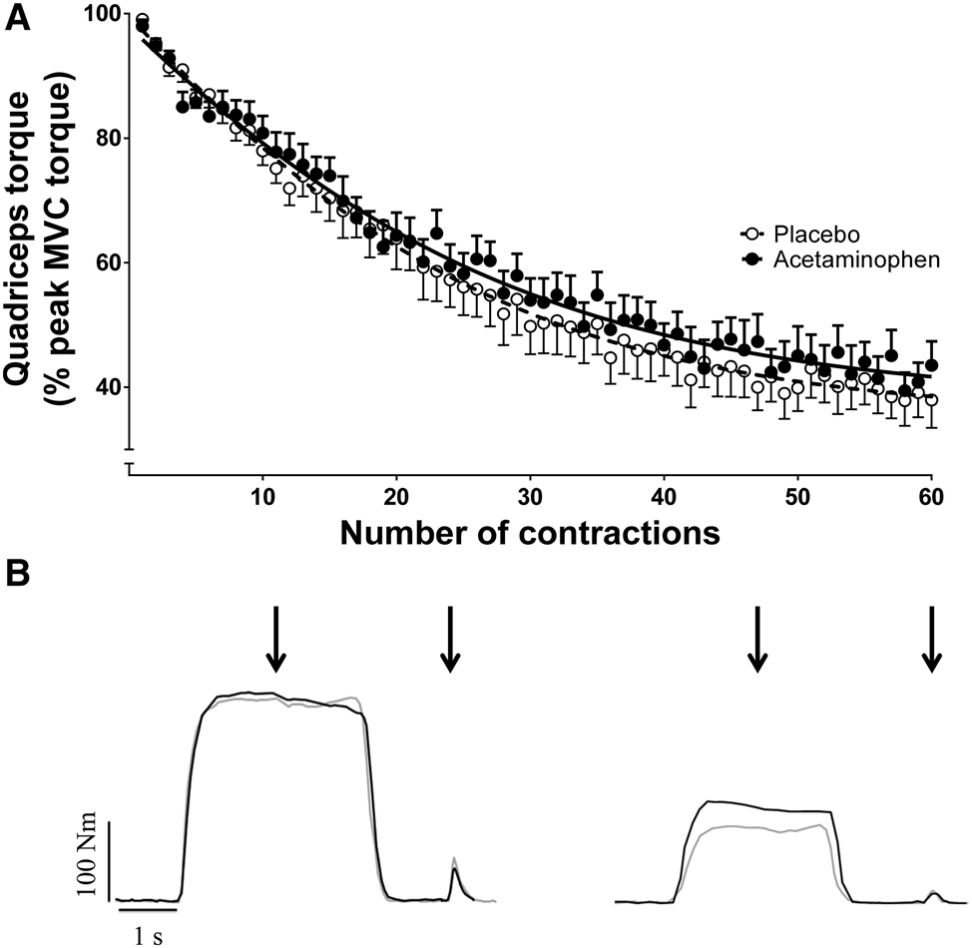
Torque profile during the 60 maximal contractions in the placebo (clear circles) and acetaminophen (filled circles) trials. All contractions were normalised to a control maximal voluntary contraction (MVC) performed before the test commenced. Mean ± SE torque responses are presented in **a** with the torque response from a representative individual presented in **b** for the 6th and 54th contractions, respectively, for PL (grey line) and ACT (black line). Note that torque falls over the first ~ 150 s before reaching stable values between 240 and 300 s (the end-test torque; last 12 MVCs). For **b**, black arrows indicate point of peripheral nerve stimulation

**Fig. 3:**
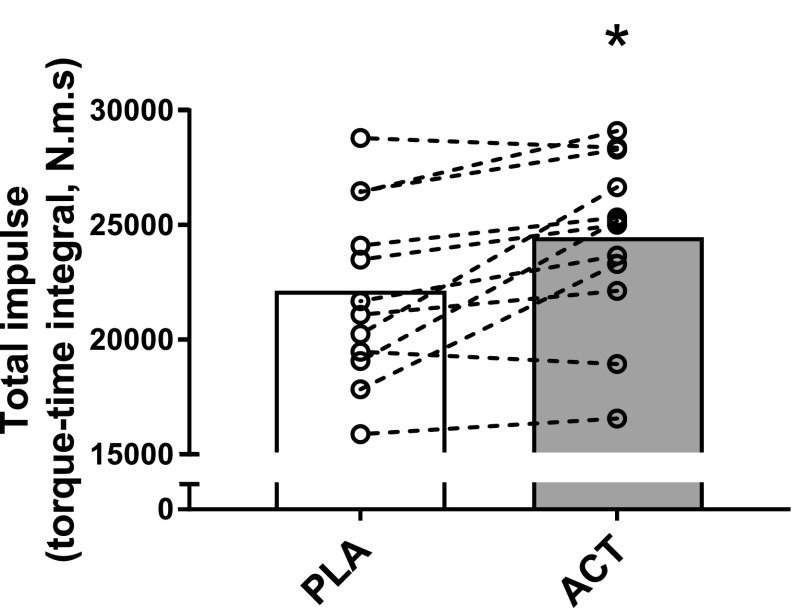
Group mean total impulse values in the placebo (PL) and acetaminophen (ACT) conditions are shown in the open and closed bars, respectively. Individual responses in the PLA and ACT conditions are shown by the open circles and linked with dashed lines. *Significantly different from PLA (*P* < 0.05)

The profile for mean torque across all subjects during each contraction for the 60 MVC protocol and a representative individual response is shown in Fig. 2. During the PL trial, torque declined from a peak of 99 ± 3% MVC (relative to pre-exercise MVC) during the first contraction to 40 ± 15% MVC during the last 12 contractions (*P* < 0.0001, *η*^2^ = 0.908; Table 1; Fig. 2). The mean torque (relative to pre-exercise MVC) achieved across the 60 MVCs was greater with ACT (61 ± 11%, 97.1 ± 23.2 N m) compared to PL (58 ± 14%, 83.8 ± 22.7 N m; *P* = 0.030, *d* = 0.656) and there was a significant interaction effect (time × condition; *P* = 0.036, *η*^2^ = 0.260). Post hoc tests revealed significant differences in torque between conditions at task end (*P* = 0.044, *d* = 0.656) but at no other time points (all *P* > 0.084 and *d* < 0.548). CT was higher with ACT compared to placebo (ACT: 44 ± 13% vs. PL: 40 ± 15%, *P* = 0.011, *d* = 0.691), with no between-condition differences in *W*^ʹ^ (PL: 6.97 ± 2.43, ACT: 6.91 ± 2.54 N m s; *P* = 0.879, *d* = 0.026). Total impulse in the 60 MVC protocol was higher with ACT (24,386 ± 3793 N m s) compared to PL (22,055 ± 3885 N m s; *P* = 0.006, *d* = 0.973). As time progressed, the difference in force production between conditions increased as evidenced by a significant difference in rates of change between conditions (i.e., difference between 300 and 150 s time points; *P* = 0.035, *d* = 0.696). The individual responses following ACT supplementation to the torque-impulse can be seen in Fig. 3. Table 2 illustrates the parameters of the 60 MVC test for PL and ACT for individual subjects and is available as an online supplement.

**Table 1:** Performance and neuromuscular function parameters of the 60 MVC test following placebo and acetaminophen ingestion

	Placebo	Acetaminophen
Performance		
Peak MVC (N m)	221 ± 49	230 ± 38
Mean torque (% MVC)	58 ± 14	61 ± 11*
Total torque-impulse (N m s)	22,055 ± 3885	24,386 ± 3792*
CT (% MVC)	40 ± 15	44 ± 12*
W^ʹ^ (N m s)	6971 ± 2432	6906 ± 2537
Neuromuscular function		
End-exercise pTw (N m)	30 ± 20	31 ± 23
End-exercise contraction time (ms)	83.2 ± 10.9	81.3 ± 14.0
End-exercise half-relaxation time (ms)	72.5 ± 17.2	75.5 ± 17.5
End-exercise MRFD (N m s)	1330 ± 471	1230 ± 433
End-exercise VA (%)	59 ± 19	62 ± 16
End-exercise M-wave amplitude (%)	96 ± 14	95 ± 18
End-exercise M-wave amplitude (mV)	5.64 ± 2.59	5.44 ± 2.72
End-exercise EMG amplitude (%)	59 ± 17	87 ± 28*

*MVC* maximal voluntary contraction, *CT* critical torque measured in the last six contractions; mean torque measured as average torque across the 60 MVC test, *pTw* potentiated twitch force, *MRFD* maximal rate of force development, *VA* voluntary activation measured using the interpolated twitch method technique, *EMG* electromyography, *N m* newton metres, *N m s* newton metres per second, *ms* milliseconds

*Significantly different from placebo (*P* < 0.05)

### Neuromuscular function

**Fig. 4:**
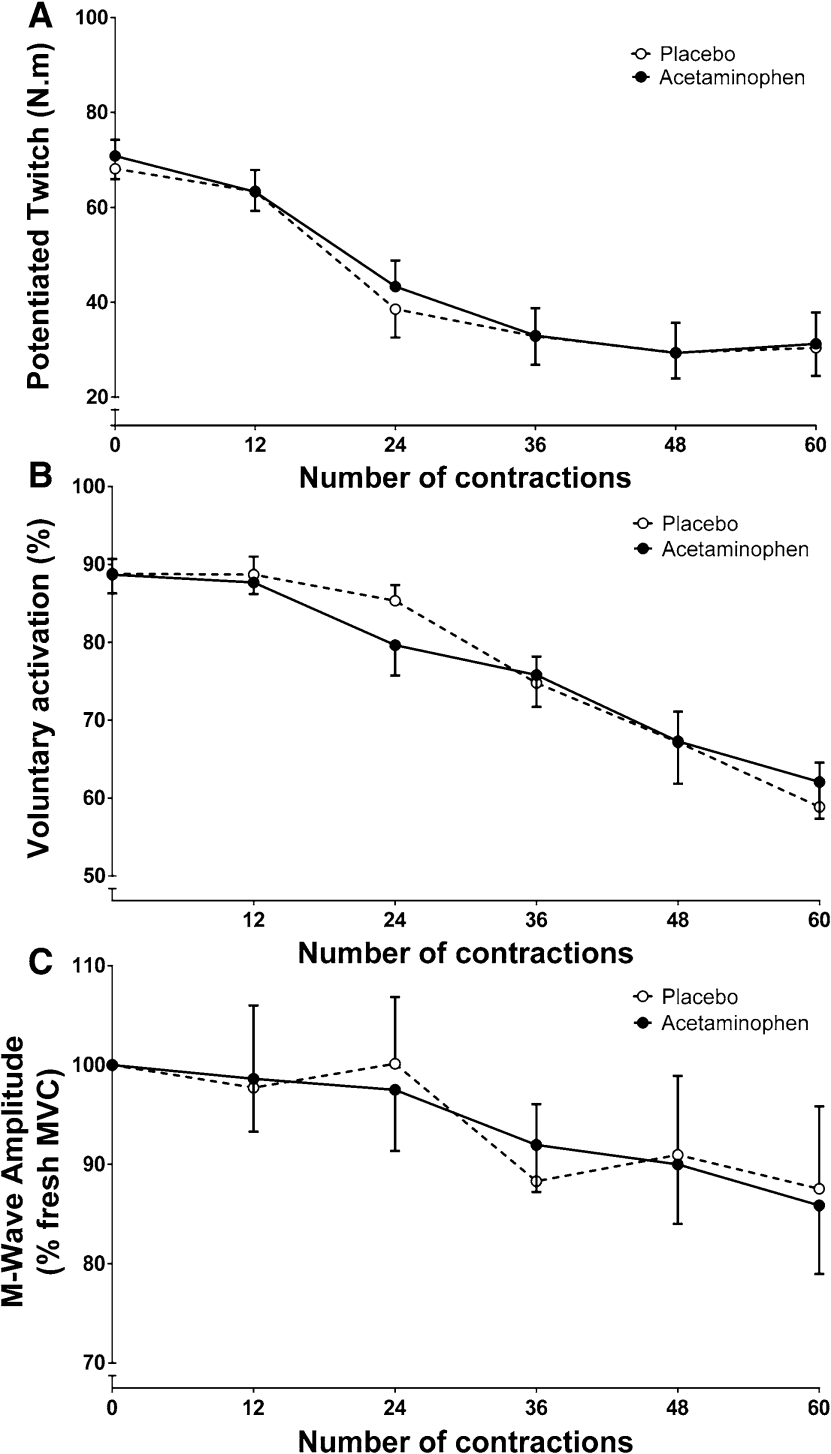
Mean ± SE potentiated twitch (**a**), voluntary activation (**b**), and M-wave amplitude (**c**) responses during the 60 maximal voluntary contraction (MVC) test for placebo (clear circles) and acetaminophen (filled circles) trials

There was a main effect for time on pTw (Fig. 4a, *P* < 0.001, *η*^2^ = 0.754), voluntary activation (Fig. 4b, *P* < 0.001, *η*^2^ = 0.647) and M-wave amplitude (Fig. 4c, *P* = 0.005, *η*^2^ = 0.281), which all declined as the protocol progressed. VA declined from 89 ± 8% to 59 ± 19% and from 88 ± 5% to 62 ± 16% in the PL and ACT conditions (*P* < 0.0001, Fig. 3), respectively. However, there was no main condition effect on M-wave amplitude (*P* = 0.733, *η*^2^ = 0.012), pTw (*P* = 0.783, *η*^2^ = 0.032) or VA (*P* = 0.841, *η*^2^ = 0.004). Likewise, there was no significant interaction effect (i.e., time × condition) on M-wave amplitude (*P* = 0.993, *η*^2^ = 0.009) or VA (*P* = 0.387, *η*^2^ = 0.097). In addition, pTw declined from 68 ± 8 to 30 ± 20 N m and from 71 ± 12 to 31 ± 23 N m (both *P* < 0.0001) for PL and ACT, respectively, but there was no significant difference in end-exercise pTw between conditions (*P* = 0.763, *d* = 0.047). There was also no significant difference in end-exercise maximal rate of force development (*P* = 0.181, *d* = 0.383), half-relaxation time (*P* = 0.234, *d* = 0.341) or contraction time (*P* = 0.595, *d* = 0.232) between conditions.

**Fig. 5:**
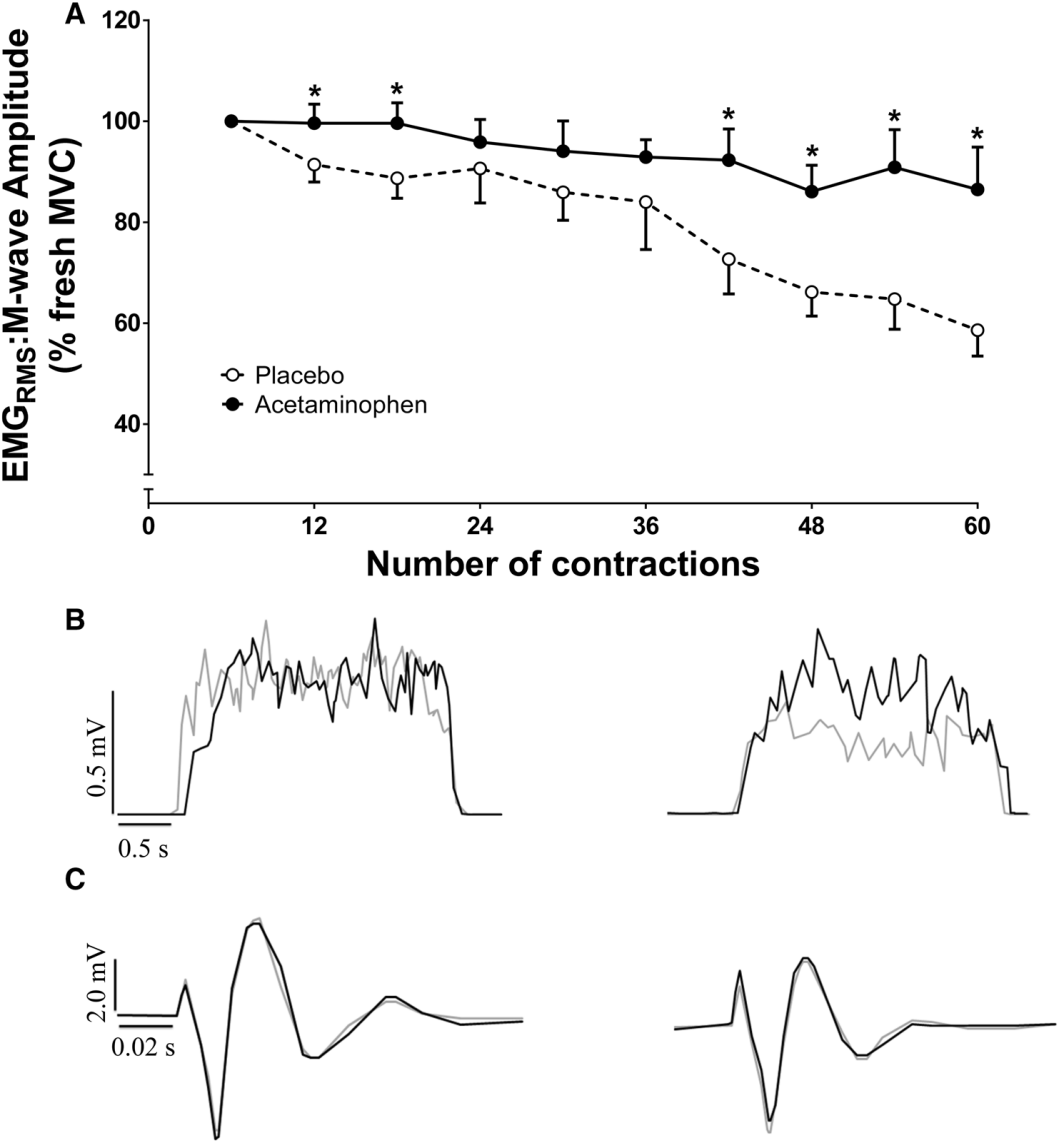
Surface electromyography (EMG) responses (expressed relative to M-wave amplitude) during the 60 MVC test for placebo (clear circles) and acetaminophen (filled circles) trials. Mean ± SE EMG responses are presented in **a** with the EMG response from a representative individual presented in **b**, for the 5th and 55th contractions, respectively, for PL (grey line) and ACT (black line). Individual representative data for M-wave can be seen in panel C for the 6th and 54th contractions, respectively, for PL (grey line) and ACT (black line). *Significantly different from placebo (*P* < 0.05)

Using six contraction (30-s) bin means, EMG_RMS_ decreased from 99 ± 4% to 59 ± 17% in the PL trial (from first six to last six contractions; *P* < 0.0001, *η*^2^ = 0.502; Fig. 5). This decline in EMG_RMS_ was attenuated following ACT ingestion (100 ± 5 to 87 ± 28%), demonstrating a significant main effect of condition (*P* = 0.033, *η*^2^ = 0.381) and an interaction effect (*P* = 0.043, *η*^2^ = 0.229). The EMG_RMS_ was elevated at 60 s (*P* = 0.036, *d* = 0.730), 90 s (*P* = 0.020, *d* = 0.835), 210 s (*P* = 0.040, *d* = 0.872), 240 s (*P* = 0.015, *d* = 0.886), 270 s (*P* = 0.019, *d* = 0.844) and 300 s (*P* = 0.001, *d* = 1.306) in ACT compared to PL (Fig. 5). As time progressed, the difference in EMG amplitude between conditions increased as evidenced by a significant difference in rates of change between conditions (i.e., difference between 300 and 150 s time points; *P* = 0.024, Cohen’s *d* = 0.804).

## Discussion

Consistent with our experimental hypotheses, the principal findings of this study were that acute ACT ingestion increased mean MVC torque, and increased CT and EMG amplitude during the latter stages of the 60 MVC protocol. These observations offer insights into the mechanisms by which ACT blunts fatigue development and suggest that the ergogenic effect of ACT ingestion may be linked to increases in CT and muscle activation during fatiguing skeletal muscle contractions.

In the present study, fatigue development and some of its underpinning mechanisms were assessed during the completion of 60 MVCs of the knee extensors using the protocol described by Burnley (2009). Consistent with Burnley (2009), torque and pTw declined by 60% and 30%, respectively, and VA declined to 59%, reflecting peripheral and central fatigue development. However, although neuromuscular fatigue was also manifest during the ACT trial, the acute ingestion of ACT increased the mean torque during the fatiguing 60 MVC protocol by ~ 5% compared to the PL condition. This finding is in line with previous observations that acute ACT ingestion can improve performance during whole body exercise in humans (Foster et al. 2014; Mauger et al. 2010).

The attenuation of neuromuscular fatigue development following ACT ingestion was not accompanied by specific alterations in central fatigue (voluntary activation), peripheral fatigue (potentiated twitch, pTw) or peripheral neuromuscular excitability (M-wave) between the ACT and placebo trials. However, while EMG declined across the 60 MVC protocol in both conditions, it declined to a lesser extent with ACT (declined to ~ 87%) compared to PL (declined to ~ 59%). This observation suggests that improved muscle activation might have contributed to the ergogenic effect of ACT ingestion. This interpretation is strengthened by our observation of a positive correlation between the inter-trial changes in EMG and torque (*r* = 0.85). Although ACT ingestion has previously been reported to increase cortico-spinal excitability at rest (Mauger and Hopker 2013), the neuromuscular mechanisms for improved performance during exercise had not been explored in previous studies reporting an ergogenic effect of ACT ingestion (Foster et al. 2014; Mauger et al. 2010, 2014). Therefore, by suggesting that ACT can improve muscle activation during repeated fatiguing skeletal muscle contractions, our findings extend previous observations by providing insight into the potential neuromuscular bases for the ergogenic effect of ACT ingestion.

It is possible that the effect of ACT may be linked to a sub-conscious neuromuscular alteration during exercise via a reduction in the magnitude of muscle afferent feedback. During exercise, ascending group III and IV muscle afferents discharge in response to noxious, metabolic and mechanical stimuli within the contracting skeletal muscles to regulate the sensation of pain, muscle activation and peripheral fatigue development (Amann et al. 2009, 2011; Blain et al. 2016; Hureau et al. 2016; McCord and Kaufmann 2010; Pollak et al. 2014). When the magnitude of type III and IV muscle afferent feedback is reduced, performance is compromised and pacing strategy is adversely impacted (Amann et al. 2009, 2011; Blain et al. 2016), despite enhanced muscle activation, as peripheral fatigue development is exacerbated. ACT administration, on the other hand, which seemingly acts predominantly through central processes to blunt pain sensation (Anderson 2008; Graham et al. 2013; Smith 2009; Toussaint et al. 2010), appears to have attenuated the decline in muscle activation without impacting peripheral fatigue development, culminating in blunted neuromuscular fatigue development in the current study. Taken together, these observations suggest that reducing, but not abolishing, pain sensation can attenuate neuromuscular fatigue development during exercise or that a higher magnitude of afferent feedback is needed to trigger a given pain sensation. Nonetheless our findings, whilst suggesting that improved maintenance of muscle activation might have contributed to the ergogenic effect of ACT ingestion, cannot differentiate between a sub-conscious alteration in neuromuscular control or a conscious ability to increase muscle recruitment via a reduction in pain sensation.

The increased muscle activation in the latter stages of the ACT trial compared to the placebo trial was accompanied by a higher mean torque over the final 12 MVCs, and therefore a higher CT (Burnley 2009). The CT is an important physiological threshold that is linked to neuromuscular fatigue development through influencing muscle metabolic homeostasis (Jones et al. 2008; Vanhatalo et al. 2016), systemic respiratory and acid-base profiles (Poole et al. 1988) and neuromuscular function (Burnley et al. 2012). During the 60 MVC protocol employed in the current study, there is a precipitous perturbation to skeletal muscle homeostasis, central and peripheral fatigue development, a decline in muscle activation and a hyperbolic reduction in torque that asymptotes at CT (Burnley 2009; Burnley et al. 2010). By lowering pain sensation (Foster et al. 2014; Mauger et al. 2010), ACT might have permitted the attainment of a greater degree of intramuscular metabolic perturbation, thereby leading to improved exercise tolerance. However, since potentiated twitch was not different between the ACT and placebo trials, it appears that ACT ingestion did not result in greater peripheral fatigue development in this study. Instead, ACT ingestion enhanced muscle activation and increased CT over the latter stages of the 60 MVC protocol. Therefore, it is also possible that ACT ingestion lowered fatigue development through permitting greater muscle activation for a given degree of intramuscular metabolic perturbation.

The blunted neuromuscular fatigue development in this study occurred in association with increased muscle activation. However, it is surprising that the attenuation in neuromuscular fatigue development was not accompanied by changes in central (i.e., voluntary activation) or peripheral (i.e., potentiated twitch force) fatigue. Indeed, since muscle activation and torque were increased, this might be expected to result in greater intramuscular metabolic perturbation and, by extension, greater peripheral fatigue development. Although we cannot exclude the possibility that ACT enhanced systemic physiological responses or skeletal muscle efficiency to mitigate this potential for greater peripheral fatigue development, we are not aware of any published evidence to support such effects.

### Experimental considerations

It is acknowledged that a limitation of the current study was that pain sensations were not directly assessed. Pain was not assessed due to the protocol requiring the completion of 60 MVCs with a short (2 s) recovery period; asking the subjects to rate pain sensation in this setting may have compromised their ability to focus on the exercise task and provide a true maximal effort during the experimental protocol. Previous studies have observed a higher power output for the same pain sensation, suggestive of a reduction in the sensation of pain during cycle ergometry exercise (Foster et al. 2014; Mauger et al. 2010). However, these studies used a higher ACT dose (1.5 g) and a different exercise modality (cycling) compared to the current study. Therefore, we are unable to draw conclusions regarding pain sensation with ACT in the current study. It should also be acknowledged that in the face of a relatively small (~ 5%) improvement in torque, it is possible that the central and peripheral fatigue measurements in our study were not sensitive enough to detect small defects in central nervous system and peripheral muscle function. Moreover, while ACT increased EMG during the latter stages of the 60 MVC protocol compared to placebo, which might be linked, in part, to increased muscle activation and central motor drive, VA was not impacted by ACT. This might have been a function of the continuous assessment of the EMG response and torque throughout the 60 MVC protocol, compared to every 6th MVC for VA, such that EMG might have provided a more complete picture of central fatigue development across the protocol. Recent research has also challenged the validity of VA estimated using the interpolated twitch technique as a measure of central fatigue (Neyroud et al. 2016), which might contribute to the disparity between the EMG-inferred central motor drive and the VA results. Accordingly, further research is required to resolve the underlying mechanisms for the ergogenic effect of ACT ingestion.

Although the current study is consistent with previous studies reporting an ergogenic effect of acute ACT consumption (Foster et al. 2014; Mauger et al. 2010, 2014), we do not advocate regular ACT use or exceeding a single dose of 1 g given the potent hepatotoxicity of ACT ingestion (Graham et al. 2013). Therefore, individuals wishing to explore the use of ACT to enhance exercise performance should do so infrequently, and with caution.

In conclusion, acute ACT ingestion increased the mean torque across 60 MVCs of the knee extensors in agreement with earlier reports that ACT can attenuate neuromuscular fatigue development and improve exercise performance. Our results extend these previous observations by providing novel insights into the mechanisms for the potential ergogenic effect of ACT ingestion. Specifically, the improved mean torque was accompanied by an increase in CT and greater muscle activation during the latter stages of the 60 MVC protocol. Therefore, ACT ingestion appears to attenuate fatigue development during repeated skeletal muscle MVCs by enabling a better preservation of muscle activation during exercise.

## Electronic supplementary material

Below is the link to the electronic supplementary material.

Supplementary material 1 (DOCX 15 KB)

## Abbreviations

ACT: Acetaminophen (paracetamol)
CT: Critical torque (i.e., asymptote of the torque-time to exhaustion relationship)
EMG: Electromyography
EMG_RMS_: EMG amplitude using root mean square method
M-wave: M-wave amplitude
*M*_max_: Maximal m-wave amplitude
MVC: Maximal voluntary contraction
PL: Placebo
pTw: Potentiated twitch
RMS: Root mean square
rTw: Resting twitch
sTw: Superimposed twitch (onto an MVC)
VA: Voluntary activation (%)
*W*^′^: Curvature constant of the hyperbolic Torque-time to exhaustion relationship
