# Supplementary material for: Acute acetaminophen ingestion improves performance and muscle activation during maximal intermittent knee extensor exercise
Source: Eur J Appl Physiol. 2018 Jan 13;118(3):595–605. doi: 10.1007/s00421-017-3794-7 (PMC5805811; doi:10.1007/s00421-017-3794-7)
Supplement: Supplementary file 1 — Supplementary material 1 (DOCX 15 KB) [file 421_2017_3794_MOESM1_ESM.docx]

| Table 2. Parameters of the 60 MVC test for placebo and acetaminophen | | | | | | | | | | | | | | |
| --- | --- | --- | --- | --- | --- | --- | --- | --- | --- | --- | --- | --- | --- | --- |
| **Placebo** | | | | | | |  | **Acetaminophen** | | | | | | |
|  | **Total**  **impulse** | **Mean**  **torque** | | **End-test**  **torque (CT)** | | **Curvature constant (Wʹ)** |  | **Total**  **impulse** | **Mean**  **torque** | | **End-test**  **torque (CT)** | | **Curvature constant (Wʹ)** |  |
| Subject | (N.m.s) | (N.m) | (% MVC) | (N.m) | (% MVC) | (N.m.s) |  | (N.m.s) | (N.m) | (% MVC) | (N.m) | (% MVC) | (N.m.s) |  |
| *1* | 19072 | 106.0 | 56.9 | 82.8 | 44.5 | 4162 |  | 25185 | 139.9 | 56.7 | 114.4 | 46.3 | 4589 |  |
| *2* | 26481 | 147.1 | 46.0 | 97.1 | 30.4 | 9008 |  | 28304 | 157.2 | 51.2 | 92.9 | 30.2 | 11591 |  |
| *3* | 17854 | 99.2 | 47.9 | 69.0 | 33.3 | 5427 |  | 23308 | 129.5 | 51.0 | 87.2 | 34.3 | 7617 |  |
| *4* | 28802 | 160.0 | 83.3 | 131.5 | 68.4 | 5124 |  | 28389 | 157.7 | 78.1 | 140.4 | 69.5 | 3123 |  |
| *5* | 21691 | 120.5 | 66.7 | 85.0 | 47.0 | 6385 |  | 23676 | 131.5 | 60.5 | 103.5 | 47.6 | 5044 |  |
| *6* | 24102 | 133.9 | 68.9 | 92.3 | 47.5 | 7495 |  | 25334 | 140.7 | 75.5 | 96.9 | 52.0 | 7899 |  |
| *7* | 23494 | 130.5 | 70.5 | 103.3 | 55.8 | 4894 |  | 25020 | 139.0 | 69.9 | 110.9 | 55.8 | 5063 |  |
| *8* | 26437 | 146.9 | 61.9 | 90.4 | 38.1 | 10166 |  | 29108 | 161.7 | 69.2 | 109.7 | 46.9 | 9353 |  |
| *9* | 19481 | 108.2 | 37.8 | 48.3 | 16.9 | 10794 |  | 18946 | 105.3 | 41.4 | 54.1 | 21.3 | 9205 |  |
| *10* | 21095 | 117.2 | 49.9 | 69.1 | 29.4 | 8662 |  | 22141 | 123.0 | 61.5 | 76.6 | 38.3 | 8356 |  |
| *11* | 20256 | 112.5 | 45.9 | 67.1 | 27.4 | 8186 |  | 26654 | 148.1 | 59.0 | 110.0 | 43.8 | 6849 |  |
| *12* | 15893 | 88.3 | 58.3 | 69.7 | 46.0 | 3349 |  | 16568 | 92.0 | 57.0 | 68.8 | 43.1 | 4178 |  |
| **Mean** | **22,055** | **122.5** | **57.8** | **83.8** | **40.4** | **6,971** |  | **24,386*** | **135.5*** | **61.0*** | **97.1*** | **44.1*** | **6,906** |  |
| **SD** | **3,885** | **22.0** | **13.7** | **22.7** | **14.7** | **2,432** |  | **3,792** | **21.1** | **10.7** | **23.2** | **12.5** | **2,537** |  |
| Mean torque, average torque achieved during the test; MVC, maximal voluntary contraction, End-test torque (i.e. CT), mean torque measured in the last 12  contractions of the 60 MVC test. *Significantly different from placebo (*P*<0.05). | | | | | | | | | | | | | | |
